# Diplopia in a patient presenting with “blurred vision”: a case report

**DOI:** 10.1186/s13256-023-04089-4

**Published:** 2023-09-08

**Authors:** Anil Harrison, Onkar Mudhar, Jun Yoo, Mayank Rampal

**Affiliations:** 1 Department of Internal Medicine, UCF/HCA, Pensacola, FL USA; 2 Internal Medicine Resident, Department of Internal Medicine, Touro University, Stockton, CA USA

**Keywords:** Blurred vision, Binocular diplopia, Ocular myasthenia gravis, Neuromuscular junction

## Abstract

**Background:**

Myasthenia gravis is an autoimmune condition affecting the neuromuscular junction and causing muscle weakness along with fatigue (myasthenia). When the clinical manifestations of myasthenia gravis are isolated to the eye muscles, only causing weak eye movements, it is referred to as ocular myasthenia gravis, which can mimic a 1 and ½ syndrome.

**Case presentation:**

An African-American female in her fifties with past medical history of hypertension presented to our outpatient clinic with complaints of blurred vision for two weeks. Her symptoms were associated with facial discomfort and a generalized headache. On physical examination upon her initial presentation, there was demonstratable swelling of the left upper eyelid with drooping. Her extraocular movements revealed defects with the abduction and adduction of the right eye, and the left eye would not adduct, although the outward movement was normal. The left eye failed to lift/elevate completely when looking upwards, a pseudo 1 and ½ syndrome. A positive Cogan lid twitch was also noticed. Imaging of the brain and orbit ruled out central causes. Diagnosis of ocular myasthenia gravis was made in accordance with positive anti-acetylcholine receptor antibodies. With 120 mg pyridostigmine oral dose, the patient experienced improvement subjectively and objectively, and the patient was discharged on oral pyridostigmine and prednisone. Six months later, with prednisone having been tapered off, the patient developed a myasthenic crisis and was treated with plasmapheresis and intravenous immunoglobulins. After recovering from the myasthenic crisis, efgartigimod infusions were instituted, which helped our patient restore normal life.

**Conclusion:**

Our patient who presented with “blurred vision” was discovered to have binocular diplopia due to significant dysconjugate eye movements. After diligently ruling out central etiologies, we concluded that her presentation was due to a peripheral etiology. Her serologies and her presentation helped confirm a diagnosis of ocular myasthenia gravis. Also, as in most cases, our patient also progressed to develop generalized myasthenia gravis while on pyridostigmine. Efgartigimod infusions instituted after our patient recovered from a myasthenic crisis have helped her restore a normal life.

**Supplementary Information:**

The online version contains supplementary material available at 10.1186/s13256-023-04089-4.

## Background

Myasthenia gravis (MG) is an autoimmune condition affecting the neuromuscular junction and causing muscle weakness along with fatigue (myasthenia). When the clinical manifestations of MG are isolated to the eye muscles, only causing weak eye movements, it is referred to as ocular myasthenia gravis (OMG). Over one-half of all patients with MG initially present with isolated ptosis, diplopia, or both, called OMG, and without any signs or symptoms of weakness elsewhere [1]. The restricted eye movements with OMG may mimic a 1 and ½ syndrome, wherein one eye does not move at all in the horizontal plane, while the other moves partially (half), usually when looking away or outwards (abduction).

Whereas, a 1 and ½ syndrome has a central etiology, caused by an insult to the brain (damage to the paramedian pontine reticular formation and/or abducens nucleus along with the medial longitudinal fasciculus on the same side) a pseudo 1 and ½ syndrome mimics a 1 and ½ syndrome, although the issue involves the peripheral nervous system. When considering a pseudo 1 and ½ syndrome, the Miller–Fisher variant of Guillain–Barré syndrome and OMG must be considered as the predominant etiologies.

Our objective is to share a video description of a patient found to have dysconjugate eye movements that developed acutely and, after ruling out important central etiologies, the near-complete resolution of symptoms and abnormal eye movements when pyridostigmine was administered. This case is also interesting since her headache, facial swelling of the left eye upon presentation, orbital cellulitis, and cavernous sinus thrombosis had also to be ruled out.

The initial approximate 27 s demonstrates significant defects with the abduction and adduction of the right eye. The right eye failed to move in the horizontal plane, and the left eye would not adduct (move inwards), although the outward movement (abduction) was normal. In addition, the left upper eyelid drooped (ptosis) and the left eye failed to lift/elevate completely when looking upwards, with a positive Cogan lid twitch noticed. [The Cogan lid twitch sign signifies that an affected eyelid will quickly rise and then fall (by as little as 1 mm or more), such that the lid appears to twitch.] After 120 mg of pyridostigmine, the subsequent sections of the video demonstrate significant improvements with ptosis and her dysconjugate eye movements.

## Case presentation

An African-American female in her fifties with past medical history of hypertension presented to our outpatient clinic with complaints of “blurred vision” for two weeks. Her symptoms were associated with facial discomfort and a generalized headache. She denied fever, problems with speech or swallowing, weakness or numbness, abnormal gait, etc. She also denied any preceding infections or vaccinations. On physical examination upon her initial presentation, there was demonstratable swelling of the left upper eyelid. Her extraocular movements revealed defects with the abduction and adduction of the right eye (the right eye failed to move in the horizontal plane), and the left eye would not adduct (move inwards), although the outward movement (abduction) was normal. The left upper eyelid drooped (ptosis), and the left eye failed to lift/elevate completely when looking upwards, a pseudo 1 and ½ syndrome. A positive Cogan lid twitch was noticed. Her vision in both eyes, a detailed fundoscopic, and a complete neurological examination were normal. Our patient was counseled for the ice pack test, but she refused it. Investigative studies to rule out emergent central etiologies with computed tomography (CT) orbits, magnetic resonance imaging (MRI), magnetic resonance angiography (MRA), and magnetic resonance venography (MRV) of the brain, along with blood work, were unremarkable. Subsequent blood work results revealed positive results of the anti-acetylcholine receptor antibodies (AChR-Ab), while anti-MUSK (antibodies to muscle-specific tyrosine kinase), and GQ1b antibodies levels were negative, as shown in Table 1. Rheumatoid factors, anti-smooth muscle antibody, anti-nuclear antibody, and anti-thyroid antibody were negative. The thyroid profile was within the normal range.

**Table 1:** Laboratory test results

Item	Result
Anti-acetylcholine receptor antibodies	Positive
Anti-MUSK	Negative
GQ1b antibodies	Negative

*Anti-MUSK* antibodies to muscle-specific tyrosine kinase,* GQ1b antibodies* Anti-Ganglioside GQ1b Antibodies

Diagnosis of ocular myasthenia gravis was made in accordance with positive AChR-Ab. Therefore, we proceeded to assess her response to pyridostigmine, and after receiving a couple of 60 mg pyridostigmine tablets, our patient experienced improvement subjectively and objectively (as demonstrated in the Additional file 1: Video). A chest CT failed to reveal a thymoma. Our patient was started on pyridostigmine 30 mg thrice a day with a gradual escalation to 120 mg four times a day, along with 10 mg of prednisone, with which our patient demonstrated a significant improvement over the next several weeks. Six months later, with prednisone having been tapered off, our patient started experiencing diplopia (double vision), significant fatigue and dyspnea, for which she was admitted and treated for the myasthenic crisis with plasmapheresis and intravenous immunoglobulins (IVIg). After recovering from the myasthenic crisis, efgartigimod infusions were instituted, which helped our patient restore normal life.

## Discussion and conclusion

Terminologies such as blurred vision used by patients might be deceiving when they might have diplopia instead. With an acute presentation of a 1 and ½ syndrome, conditions such as multiple sclerosis, cerebrovascular accident, infection, trauma, tumor, and conditions involving the brain must be ruled out. Ocular myasthenia gravis (OMG) and Miller–Fischer syndrome (MFS), a variant of Guillain–Barré syndrome are peripheral etiologies for diplopia and are referred to as a pseudo 1 and ½ syndrome. When areflexia and ataxia accompany ophthalmoplegia, one needs to consider a diagnosis of MFS [2, 3]. Antibodies against GQ1b are present in most patients with MFS [4].

Muscle weakness due to dysfunction of the neuromuscular junction (myasthenia), mediated by autoantibodies, is usually an acquired disorder and is referred to as myasthenia gravis. Interestingly, 50% of all patients with MG present initially as ocular myasthenia gravis [2, 5]. Diplopia, secondary to extraocular muscle weakness, is a prominent feature of OMG. The weakness can involve either a single or multiple extraocular muscles, in one or both eyes. Although the ice pack test is not diagnostic, it can be used as part of the neurologic examination for patients with drooping eyelids (ptosis). When suspecting OMG, the application of an ice pack to the upper eyelid helps improve the ptosis. The test is based on the physiologic principle that neuromuscular transmission improves at lower muscle temperatures and hence, in patients with MG, ptosis can be overcome temporarily by direct cooling of the eyelid muscles. The sensitivity appears to be approximately 80% in those with prominent ptosis due to MG [6]. The Edrophonium (Tensilon) test is no longer used when evaluating myasthenia gravis. When considering a diagnosis of OMG, a positive ice pack test might help raise one’s suspicion, following which checking for anti-acetylcholine receptor antibodies would be the next step, which, if positive, confirms a diagnosis of OMG. Checking for muscle-specific tyrosine kinase antibodies (MUSK) is also reasonable. If these tests are negative, and the suspicion is high, electromyography with the rapidity of nerve stimulation (RNS) or single fiber electromyography (SFEMG) would be the next steps to perform. When serum antibody testing and electromyography do not help clinch the diagnosis, further testing requiring imaging of the central nervous system along with spinal fluid analysis might be required to exclude central nervous system structural disorders and inflammatory conditions. To help alleviate the symptoms of diplopia, either an eye patch or specialized lenses can be used. Although pyridostigmine, an anticholinesterase, is used most often in the treatment of myasthenia gravis, it rarely helps with diplopia secondary to OMG. It is recommended to be used in mild cases of OMG or as an adjunctive symptomatic treatment for moderate or severe OMG. Our patient interestingly had a significant improvement with her diplopia for some months. Immunosuppressive treatment is recommended when OMG is severe, although its usage has been controversial. Prednisone is used quite commonly to provide symptomatic benefits, while interestingly some studies even suggest treatment with corticosteroids may reduce the progression to generalized myasthenia gravis (GMG) [7]. When patients with OMG do not respond to prednisone, second-line immunosuppressants such as mycophenolate mofetil, azathioprine, rituximab, and cyclosporine can be used. Efgartigimod alfa, a neonatal Fc receptor antagonist, is an IgG antibody Fc-fragment molecule designed to promote the degradation of IgG autoantibodies [8] that can be used for patients who are acetylcholine receptor antibody positive. Thymectomy is required for patients who have a thymoma and myasthenia, although it can be a consideration even in patients with GMG who do not have a thymoma since several case series have demonstrated a similar response to thymectomy in OMG as with GMG [9].

A similar presentation may occur with Graves’ disease, which causes a constrictive ophthalmopathy. However, it can be differentiated from MG by the lack of ptosis. Other features found in Graves’ disease such as proptosis, lid retraction, lid lag, and periorbital edema are not seen with OMG. The other differential diagnoses for OMG are chronic conditions that progress slowly, such as “chronic progressive external ophthalmoplegia and Kearns–Sayre syndrome,” both of which are mitochondrial disorders. Some other differential diagnoses include myotonic dystrophy, oculopharyngeal dystrophy, and structural diseases of the brainstem such as aneurysms and parasellar tumors.

In conclusion, our patient who presented with “blurred vision” was discovered to have binocular diplopia due to significant dysconjugate eye movements. After diligently ruling out central etiologies, orbital cellulitis, and cavernous sinus thrombosis, we concluded that her presentation was due to a peripheral etiology. Her serologies and her presentation helped confirm a diagnosis of OMG. Also, as in most cases, our patient also progressed to develop generalized myasthenia gravis while on pyridostigmine. Efgartigimod infusions instituted after our patient recovered from a myasthenic crisis have helped restore a normal life.

### Supplementary Information

**Additional file 1: **Dysconjugate eye movements resolved with Pyridostigmine.

## Data Availability

All data generated during this study can be accessed through direct communication with the corresponding author and the agreement of all research team members.

## Abbreviations

MG: Myasthenia gravis
OMG: Ocular myasthenia gravis
CT: Computed tomography
MRI: Magnetic resonance imaging
MRA: Magnetic resonance angiography
MRV: Magnetic resonance venography
AChR-Ab: Anti-acetylcholine receptor antibodies
anti-MUSK: Antibodies to muscle-specific tyrosine kinase
IVIg: Intravenous immunoglobulins
MFS: Miller–Fischer syndrome
RNS: Rapidity of nerve stimulation
SFEMG: Single fiber electromyography
GMG: Generalized myasthenia gravis
